# Genetics in primary care: validating a tool to pre-symptomatically assess common disease risk using an Australian questionnaire on family history

**DOI:** 10.1186/s40169-019-0233-x

**Published:** 2019-05-02

**Authors:** Elisa J. F. Houwink, Olga R. Hortensius, Kees van Boven, Annet Sollie, Mattijs E. Numans

**Affiliations:** 1 Department of Public Health and Primary Care (PHEG), Leiden University Medical Center, Hippocratespad 21, Building 3, V6-54, 2333 ZD Leiden, The Netherlands; 2 Department of Primary and Community Care, Radboud University Medical Center, PO Box 9101, 6500 HB Nijmegen, The Netherlands; 3 Maison de Santé Bel Air, 154 Avenue Bel Air, 84200 Carpentras, France

**Keywords:** Family history questionnaire, Genetics, Primary care, Qualitative study, Face and content validity

## Abstract

**Background:**

A positive family history for diabetes, cardiovascular diseases or various types of cancer increases the relative risk for these diseases by 2 to 5 times compared to people without a positive family history. Taking a family history in daily general practice is useful for early, pre-symptomatic risk assessment, but at the moment no standardized family history questionnaire is available in the Dutch language. In this study we used a 9-item questionnaire, previously developed and applied in an Australian study, to probe family history for 7 specific conditions. The aim of the present qualitative study was to test face and content validity of the Australian family history questionnaire in Dutch general practice and to advance the standardization of intake information at an international level. We conducted 10 cognitive interviews with patients over 4 rounds, using the verbal probing technique. This approach allows the collection of data through a series of probe questions, with the aim of obtaining detailed information. After each interview round we modified the questionnaire based on the answers of the interviewees. We also performed 10 semi-structured interviews with general practitioners (GPs) to get their opinion on the content and usability of the questionnaire in practice.

**Results:**

Patients varied in age and gender, and 4 patients were known to have a genetic disorder. The GPs varied in age, gender, clinical experience, type of practice and location. In the first round, seven problems were identified in the questionnaire in the categories *Comprehension (1), Recall (2), Judgement (0), Response process (2)* and *Completeness*, *(2)*; by the fourth and final round no problems remained. The content and usability of the questionnaire were assessed positively.

**Conclusions:**

When translated for everyday use in Dutch general practice, the Australian family history questionnaire showed a strong face and content validity, and GPs were positive regarding feasibility. Validation of this family history questionnaire could aid in the standardized integration of genetically relevant information in the electronic health record and clinical research. Conspicuous questionnaire information might alert the GP regarding specific conditions and enable detection of disease at an earlier stage. Additional questionnaire requirements needed however are accurate patient information and consistent, accessible locations in the electronic health record with a possibility to be automatically registered. By deriving a Dutch family history questionnaire convenient for GPs, we adapted a template that might also prove useful for other countries and other medical professionals. This development could make the rapid operationalization of readily available genetic knowledge feasible in daily practice and clinical research, leading to improved medical care.

## Background

A positive family history for diabetes, cardiovascular diseases or various types of cancers, including prostate cancer, ovarian cancer, melanoma, breast cancer and colon cancer, leads to a relative risk for these diseases two to five times higher than that of people without a positive family history, irrespective of known genetic associations (e.g. *BRCA1/2*). When multiple family members are affected with these common diseases, and when this occurs at a young age, the relative risk increases further [1, 2]. A family history is therefore a useful tool for pre-symptomatic risk assessment for multiple common chronic diseases (e.g. cardiovascular diseases, cancer, diabetes) in daily primary care practice, reinforced by the role of GPs as family doctors. A family history could open possibilities for early primary and secondary prevention of these diseases and their monogenetic disease equivalents (e.g. long QT syndrome, breast cancer caused by *BRCA1/2* mutations, MODY subtypes) and could also be used to find, inform and treat unaffected family members pre-symptomatically [3]. In a qualitative Dutch study, general practitioners (GPs) recognized the urgent need for competence in family history taking and in the registration of a questionnaire in the electronic health record (EHR), yet GPs mentioned a lack of knowledge regarding genetics [4]. Nevertheless, GPs agreed that taking a family history using a validated questionnaire could be an important tool in good clinical practice as it allows for familial risk stratification and the identification of hereditary conditions [1, 4–6]. In addition, it was suggested that a validated online family history questionnaire could aid in the decision-making process (decision support systems) surrounding the consultation of a clinical geneticist for further diagnosis in accordance with current clinical genetics referral guidelines [7]. A validated family history questionnaire would therefore help integrate genetics into the EHR, leading to the rapid operationalization of readily available genetic knowledge in daily practice and clinical research, consequently improving medical care.

On the other hand, Acheson et al. showed that GPs do not always discuss family history with their patients. Family issues were discussed in about half of all entry visits by new patients and in only 22% of visits of previously enlisted patients [2]. These figures may even be an overestimate, as family issues also include topics other than family history alone. Furthermore, family history is not always (adequately) recorded in electronic health records [7–9], and Dutch GPs have indicated that electronic health records do not have retrievable codes for family history [6–8]. A frequently mentioned barrier to the taking of a family history is the lack of time, as GPs have only 10 min per patient and usually have other priorities [10]. Therefore, most GPs update a family history only when necessary. A concise family history questionnaire could help remove this barrier. However, there is currently no standardized approach to the taking of a family history in primary care and GPs almost never use a formal questionnaire or tool for a family history [4–6]. In a Belgian study, GPs indicated they would prefer a tool for a structured family history [5].

A family history questionnaire was recently developed by Emery et al. [11] for Australian primary care. The questionnaire consists of 15 questions, three of which cover ethnicity, while the remaining 12 cover conditions or illnesses found in a family. Of these 12 questions, nine discuss seven specific conditions: cardiovascular disease, diabetes, prostate cancer, ovarian cancer, melanoma, breast cancer and colon cancer. The study showed that these nine questions can accurately screen for increased risk for the conditions included [11]. Using the same questionnaire, an implementation study by Reid et al. stated that the questionnaire could be easily completed [12].

In the Netherlands, as in many other countries, there is currently no standardized family history questionnaire. Our aim was to develop a Dutch family history questionnaire that could be used easily by GPs and that could act as a template for other countries in their standardization of entry information on family history in primary care practice [13]. Our family history questionnaire consisted of the 9 disease-oriented questions developed in the Emery et al. questionnaire, but translated into Dutch by a network of GPs in Nijmegen (FaMe-net) [14]. The complete interview guides and questionnaire on family history can be found in Appendices.

## Methods

### Aim

Using an Australian family history questionnaire translated into Dutch, in this qualitative study we aimed to test the face and content validity of the questionnaire in Dutch primary care practice through analysis of interviews with patients and GPs.

### Design

This qualitative study used face-to-face cognitive interviewing methods to interview patients and semi-structured interviews to interview GPs. The interviews focused on the family history questionnaire (Appendix 1).

### Cognitive interviewing

Cognitive interviewing is a technique that evaluates sources of response error to a questionnaire [15]. There are two approaches to cognitive interviewing: think-aloud interviewing and the verbal probing technique. We chose the verbal probing technique, which is a more active form of data collection in which the cognitive interviewer administers a series of probe questions specifically designed to elicit detailed information beyond that normally provided by respondents [15, 16].

Verbal probing is based on a model developed by Tourangeau and Rasinski [17]. The model consists of four processes: comprehension of the question, retrieval from memory of relevant information (recall), decision process (judgement) and response process (Table 1) [18]. We used retrospective probing, a technique in which the interviewer administers the probe questions after the respondent has completed the entire questionnaire [19].

**Table 1:** Examples of cognitive interviewing according to Tourangeau and Rasinski [17]

Cognitive probe	Explanation	Example of interview question
Comprehension	What does the respondent believe the question to be asking?	Can you repeat the question in your own words?
Recall	What types of information does the respondent need to recall in order to answer the question?	How did you arrive at your answer?
Decision/judgement process	Does the respondent devote sufficient mental effort to answer the question accurately and thoughtfully?	How hard was it to answer the question?
Response process	Can the respondent match his or her internally generated answer to the response categories given by the survey question?	Did you wish to give an answer different to the available answer options?

### Setting of the study

The setting of the study was Dutch primary care.

### Recruitment and participants

Participants were recruited by approaching GPs in and around the western part of the Netherlands by email or personally through the network of the Department of Public Health and Primary Care at Leiden University Medical Centre. GPs were asked to participate in a study in which (1) they themselves would be interviewed, and (2) would invite some of their patients to be interviewed. We then contacted patients who were willing to participate. Patients were also recruited by means of flyers, posters and information leaflets. We used purposive sampling to maximize variance in characteristics.

#### Characteristics of participants

We conducted 20 interviews, 10 with GPs and 10 with patients.

### Data collection and analysis

One interviewer conducted all interviews (OH), in the Dutch language. The patient interviews were conducted at a patients’ home or at the LUMC and lasted for approximately 20 min. The GP interviews were conducted at the LUMC or at a GP’s practice, and also lasted for approximately 20 min. The interviewer used both scripted and spontaneous probes. The complete interview guides can be found in Appendices 2 and 3. All interviews were audio-recorded and transcribed verbatim. Before the interview, participants were asked to fill out a questionnaire with sociodemographic questions in order to define population characteristics (Appendices 4, 5).

**Fig. 1:**
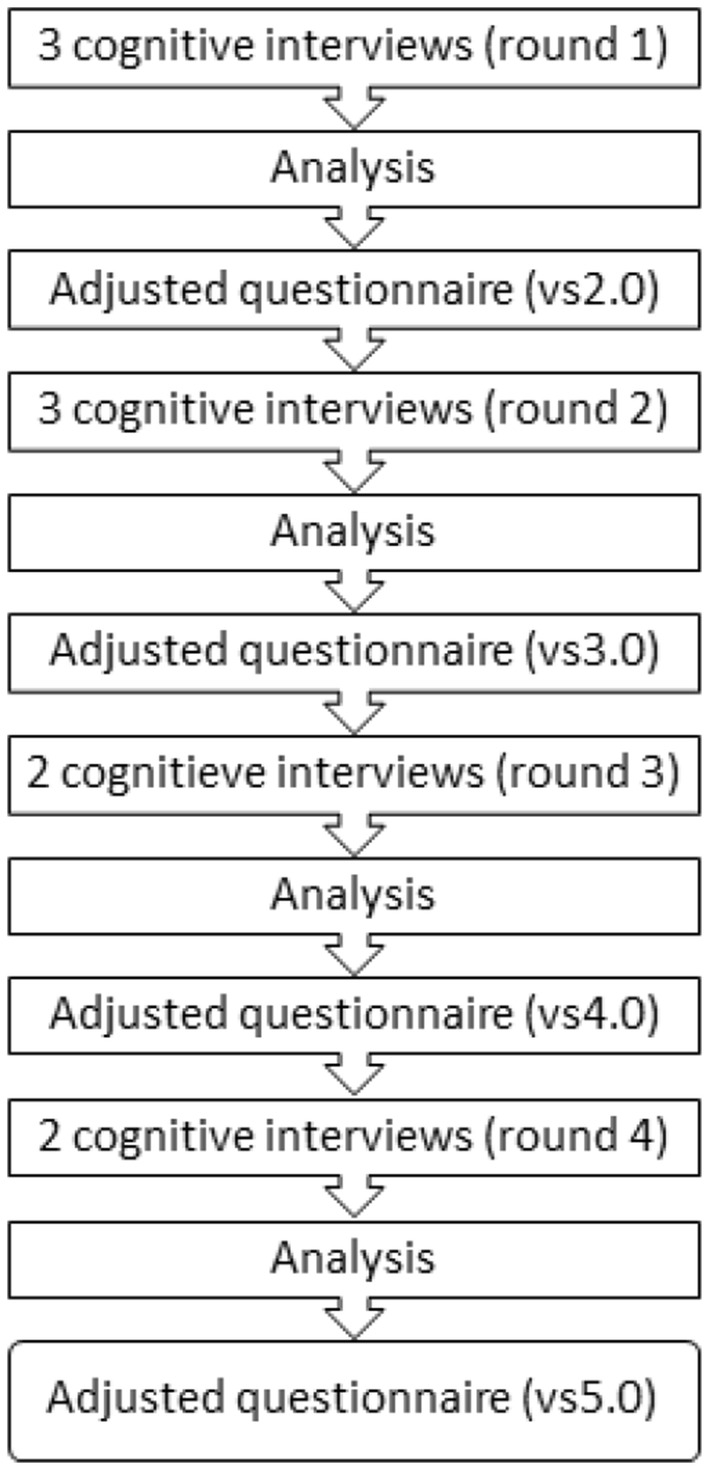
Data collection and analysis

The patient interviews were analysed using the following categories based on the Tourangeau and Rasinski [17] model: comprehension, recall, judgement, response process and completeness. This allowed analysis of the respondents’ cognitive process [15]. The patient interviews were conducted in four rounds (2 or 3 interviews per round). After each round, problems in the questionnaire were identified and, where necessary, the questionnaire was modified. In the next round of interviews, the modified version of the questionnaire was used (see Fig. 1). The GP interviews were analysed using conventional content analysis to identify the themes in the interviews [19]. Two researchers coded the transcripts of the interviews independently (OH and EH). Each participant was interviewed once, so the rounds were with different participants. Atlas.ti was used for analysis of the interviews. Data saturation was reached when no new information could be derived from GP interviews and no further problems could be identified in the patient interviews.

### Ethics

Participants gave written informed consent prior to being interviewed. The Medical Ethics Committee of Leiden University Medical Centre (LUMC) approved the study (reference number P18.057).

## Results

### Patient characteristics

Mean patient age was 49.5 years (range 26 to 74 years) and most participants were female (7 out of 10). Three patients had received higher education, 6 patients middle level education and 1 patient a lower level education. Six patients had no known genetic disorder, while 4 patients had a known genetic disorder. Four patients were in remunerative employment, 2 were students, 2 were unemployed, 1 was retired and 1 was incapacitated. An overview of patient characteristics is presented in Table 2.

**Table 2:** Characteristics of patients

No.	Age	Gender	Level of education^a^	Employment	Genetic disorder	Round of analysis
1	35	Female	Middle	Incapacity for work	Yes	1
2	30	Female	Higher	Remunerative	Yes	1
3	55	Female	Middle	Remunerative	No	1
4	26	Female	Middle	Student/remunerative	No	2
5	74	Male	Middle	Retired	Yes	2
6	63	Female	Middle	Remunerative	No	2
7	69	Female	Lower	Unemployed	Yes	3
8	62	Male	Middle	Remunerative	No	3
9	26	Male	Higher	Unemployed	No	4
10	55	Female	Higher	Student/remunerative	No	4

Level of education divided into lower (no education, primary education, lower secondary education or lower vocational education), middle (secondary vocational education or high school) and higher (higher professional education or university) [18]

### Characteristics of general practitioners

The GP’s mean age was 51.1 years (range 33 to 62 years), with a mean of 19.4 years (range 3 to 37 years) clinical experience. Seven GPs were female and 3 male. The type of practice was relatively evenly distributed as 5 worked in a group practice, 3 in a duo practice and 2 worked in a solo practice. Four were GP partners, 3 were sessional GPs (defined by the National Association of Sessional GPs (NASGP) as a locum GP, a fully qualified independent general practitioner who does not have a standard employment contract with the primary care health center where they work. They are paid by hour of work), 2 were non-practicing and 1 GP was in training. The non- practicing GPs were recruited through the network of the Department of Public Health and Primary Care at Leiden University Medical Centre. These GPs recently stopped as practicing GPs with many years of clinical experience, but are still working for the Department of Public Health and Primary Care in Leiden. Four of the GPs worked in an urban area, while 6 worked in a rural area. An overview of all GP characteristics is presented in Table 3.

**Table 3:** Characteristics of general practitioners

No.	Age	Gender	Clinical experience	Type of GP	Type of practice	Location
1	36	Female	5	Sessional GP	Solo	Rural
2	60	Male	25	Non-practicing GP	Group^a^	Rural^a^
3	58	Female	20	GP partner	Duo	Rural
4	45	Male	15	Sessional GP	Group	Rural
5	57	Female	30	Sessional GP	Solo	Rural
6	57	Female	20	GP partner	Duo	Urban
7	33	Male	3	GP in training	Group	Urban
8	59	Female	25	Non-practicing GP	Duo^a^	Rural^a^
9	44	Female	14	GP partner	Group	Urban
10	62	Female	37	GP partner	Group	Urban

^a^The last practice worked

### Results of patient interviews

The interviews were analysed using the following categories based on the model by Tourangeau and Rasinski [17]: comprehension, recall, judgement, response process and completeness [16–19]. Problems in the questionnaire were identified and modified accordingly before the following round. An overview of the identified problems can be found in Table 4. The problems identified and the changes made are described per category. The final version (v5.0) of the questionnaire can be found in Appendix 6, with the modifications in *italics*. The categories are described below, with patient quotes in bold.

**Table 4:** Identification of problems in the questionnaire

Category	Round 1	Round 2	Round 3	Round 4
Comprehension	More than one (1)	On the same side of the family (1) Type 1 or type 2 (1) Close relatives (1)	Age limits (1) Same side of the family (1)	–
Recall	Loss of contact (2)	–	–	–
Judgement	–	–	–	–
Response process	Missing category (2)	–	–	–
Completeness	Open question (2)	Open question (2)	–	–

The number of patients who had a problem with an item is given in parentheses

### Comprehension

Based on the patient interviews, in round 1 the term ‘diabetes’ (question 2) was replaced with the Dutch word for diabetes (“suikerziekte”), since it was thought that this would be easier for patients to understand. One participant in round 1 misinterpreted questions 5 and 9. Although both questions referred to ‘more than 1 family member’, the participant included only one family member. Therefore, ‘more than 1 family member’ was changed to ‘2 or more family members’. In round 2 one participant did not notice the phrase ‘on the same side of the family’ in questions 5 and 9. To emphasize this phrase, ‘on the same side of the family’ was subsequently underlined. In round 2 one participant mentioned that a family member had diabetes at an older age but did not realize that this was equivalent to type 2 diabetes. Therefore ‘(type 1 or type 2)’ was removed from question 2.

Female, aged 63, while filling in the question about diabetes: *“Yes, diabetes at an older age, that is not hereditary, right? If one of my parents, brothers or sisters, type 2, what is type 2? No, not really, No.”*

Another participant was confused as to which family member the question was referring to. Therefore, ‘relatives’ was changed to ‘close relatives’ in questions 1, 2, 3, 4, 6, 7 and 8. In round 3 one participant didn’t notice the age limits in questions 1, 4, 6 and 8. To add emphasis, age limits were underlined. One participant in round 3 didn’t understand what was meant by ‘on the same side of the family’, therefore ‘(father’s/mother’s side)’ was added after ‘on the same side of the family’.

### Recall

In round 1 two participants could not answer questions 5 and 9 completely, due to loss of contact with aunts, uncles or cousins. A response category ‘not to my knowledge’ was added to overcome this problem.

Female, aged 35, about question 5: *“Say, uncles, aunts, cousins and grandchildren, maybe you don’t know that. That there maybe should be an option ‘not to my knowledge’ or something like that.”*

Other problems mentioned by participants were difficulties in remembering exactly when an older family member became ill when still young, and that in the past illness was less often discussed than it is today. Another problem in the category recall was that two participants did not know the answer to the questions 5 and 9, which is further explained in the category “Response process”.

### Judgement

In the category ‘Judgement’ no problems were identified in the questionnaire. One participant mentioned that some questions seemed to be a little ‘emotionally charged’, but this did not hinder answering the questions.

### Response process

As described in the category ‘Recall’, two participants in round 1 could not completely answer questions 5 and 9. To overcome this problem the answer category ‘not to my knowledge’ was added.

### Completeness

In rounds 1 and 2, four participants mentioned that they wished to report another condition found in the family. Therefore, an open question was added to allow the reporting of other diseases.

### Other

Most of the participants (9/10) found the questionnaire to be concise and easy to complete. Only one participant thought that the questionnaire was too long and remarked that 6 or 7 questions would have been better than 10.

*Male, aged 26: Interviewer: “And in general, what did you think of the number of questions?”*

*Participant: “I turned the paper over as I thought there would be another list; questionnaires usually take quite a long time, so I thought it was easy.”*

Most participants were positive regarding their likely response to receiving the questionnaire from their GP and stated that they would complete the questionnaire. Only one participant stated that he would be unwilling to complete the questionnaire for a GP, as he was concerned about privacy issues and did not want the information in his EHR.

*Female, aged 30: Interviewer: “And what would you think if you received this questionnaire from your GP?”*

*Participant: “Actually, it is perfectly logical. I think it is important that your doctor should have this information. Certainly, yes.”*

### Results of interviews with general practitioners

The interviews with GPs were analysed using conventional content analysis. Two main themes surfaced during the interviews: ‘the content of the questionnaire’ and ‘the use of the questionnaire’. There were also subthemes within both themes. The subthemes for ‘the content of the questionnaire’ were ‘terminology’, ‘length of the questionnaire’, ‘formulation of the questions’, ‘completeness’ and ‘structure’. The subthemes for ‘the use of the questionnaire’ were ‘current use of family history’, ‘usefulness of the questionnaire’ and ‘barriers’. The themes are described below, with quotes from GPs in grey boxes.

### The content of the questionnaire

#### Terminology

Three specific words in the questionnaire elicited comment from most GPs. First, regarding the use of the word “diabetes (type 1 or type 2)”, most GPs thought the more commonly used Dutch word for diabetes would be easier to understand. Some GPs also thought that “type 1 or type 2” would be confusing, as not everyone would understand the meaning.

The second word GPs debated was “heart disease”. GPs felt this to be a rather broad term that includes multiple conditions such as atrial fibrillation, other arrhythmias, heart failure, heart attacks and hypertension. Some GPs stated that many patients would only know that a family member had “something wrong with their heart” or is “a heart patient”, but they would not know the exact condition.

The final word that the GPs thought would be difficult for patients to understand was “melanoma”. They felt that not all patients would recognize the term and found the accompanying explanation in parentheses, “a malignant mole”, clearer. Another concern was that patients would include other types of skin cancer in answer to this question because they might not know the difference between melanoma, basal cell carcinoma and squamous cell carcinoma.

#### Length of the questionnaire

Almost all GPs were happy with the length of the questionnaire. They stated that a questionnaire should not include more than 10 questions and be no more than one page. One GP thought the length of the questionnaire might have a negative impact on patients, as patients might start worrying about risks for diseases included in the questionnaires. One GP thought the questionnaire length was fine for the more literate but might be too long for the poorly literate.

*Female GP, aged 62: “Yes, it is quite long; I think you have to make a distinction between people with a reasonable education who can read well, in which case it is fine to ask this many questions, it is not that difficult. But for people who are poorly literate, it is a lot.”*

#### Formulation of the questions

Most GPs thought the formulation of the questions was fine. Two GPs misinterpreted a question because they misread the question. In questions 5 and 9 they failed to notice the phrase “more than 1”, but when this was included they better understood the question. However, they did suggest that “more than 1” should be changed to “2 or more”.

Some GPs commented that questions 5 and 9 were too long, mostly due to the mention of family members in parentheses. They thought this might make the question harder for some patients to understand.

#### Completeness

Some GPs missed an open question concerning rare hereditary conditions. Cerebrovascular Accident (CVA) was mentioned twice as missing from the questionnaire. Other conditions mentioned by one GP included high cholesterol and mental disability. One GP missed a question about consanguinity.

*Male GP, aged 45: “I think you have all the big ones, but if you also want the rare syndromes you could miss those. How you would phrase it is of course very difficult, but there are families with something very rare but very serious. Do you want to have some sort of additional category? That is the only consideration, but all in all, the common diseases are all there.”*

When GPs were asked if they felt certain conditions were overlooked in the questionnaire they stated that their knowledge of genetics was insufficient to answer the question properly.

#### Structure

A few GPs mentioned changing the structure of the questionnaire. One GP thought that the questionnaire would be better if it took the form of a flowchart starting with a question regarding “an illness running in the family”. If the answer was “No” the patient could stop at that point, saving a considerable amount of time. Two GPs suggested changing the order of the questionnaire, with questions ranked in order of risk or prevalence. Other GPs mentioned the use of sub-phrases for questions 5 and 9, in view of the long sentence structure. They suggested first asking whether breast cancer ran in the family, and if so, who exactly had it and at what age.

### Use of the questionnaire

#### Current use of family history

8 out of ten GP participants clarified during the interviews they only raise the issue of family history when a patient consultation is in relation to a previously diagnosed condition, similar to previously found qualitative research results on this topic [4, 5]. In other words, family history is still used passively rather than proactively, even though it is confirmed it could prevent hereditary forms of disease or detect these diseases in an early phase.

In some GP practices, mainly younger GP participants, an intake form was used when patients registered with the practice, which sometimes included a few questions about family history. These questions were generally not as detailed as the questions in the questionnaire. The GPs would then discuss the patient’s answers during an introductory meeting. These results confirm the results found previously by Houwink et al. and Daelemans et al. [4, 5].

*Interviewer: “Did you often discuss these questions with patients?”*

*Male GP, aged 60: “No, well, with a reason. But I have to be honest; I think mostly I was reasoning backwards. If somebody had something then you would think, ‘Oh, perhaps it runs in the family?’”*

#### Usefulness of the questionnaire

Most GPs agreed that the questionnaire provides useful information to prevent disease. One GP even mentioned that she had felt a need for this type of questionnaire. GPs also agreed that the information would be useful for prevention, mentioning both primary and secondary prevention. When a GP knows that a patient is at risk, for example for cardiovascular disease, they could advise lifestyle interventions to prevent cardiovascular disease. If questionnaire information was conspicuous in the EHR, a GP would be more alert regarding specific conditions and better able to detect disease at an earlier stage. Furthermore, regular use of the questionnaire at appropriate intervals would help collect information on a patient’s family members who had developed disease in the intervening years or had a previously undetected condition, which is indicated by the following statement:

*Female GP, aged 57: “Well, when people have symptoms you make a different risk assessment. You are more alert. Because if it’s (questionnaire information) there and somebody is coming in, it says that something runs in the family and then you think, ‘Oh, that was that family, well, let’s have a look’. So it’s kind of an extra alarm bell.”*

Some GPs felt there were additional requirements before the questionnaire could be considered useful. One requirement was a consistent location in the EHR where positive answers to questionnaire items could be registered. If this was not possible, the information would be inaccessible and would be of little use in the early detection of disease. Another requirement was that the questionnaire information provided by the patient should be accurate. Some GPs suggested that a positive answer to a question in the questionnaire should be followed by a doctor-patient talk to obtain more information. During this talk the GP could judge the reliability of the information, which they could then use for better risk estimation.

A third requirement mentioned by some GPs was that a patient should receive a clear information leaflet explaining the purpose and value to the GP of the questionnaire. Understanding the usefulness and personal benefits would help motivate patients to take the time to complete the questionnaire.

#### Barriers

Some GPs mentioned certain barriers to use of the questionnaire. Two GPs mentioned the considerable time needed to complete and register all patient questionnaires in the EHR, although one of these GPs agreed that the questionnaire would be useful. The other GP felt that the health benefit for the patient would not balance the time needed. Another GP stated that it would be useful to have the questionnaire registered automatically in the EHR. Around half of GPs had questions related to the implementation of the questionnaire. Questions such as: “When would the questionnaire be filled in?”, “Will you send the questionnaire to all patients?”, “How are you going to sell this to patients”, “How often do we give the questionnaire?” and “How are you going to implement this?” GPs thought it would be easier to let new patients complete the questionnaire at intake than to ask current patients to retrospectively complete the questionnaire. One GP suspected it would be hard to convince patients to take the questionnaire. Another GP, with experience of other questionnaires, expected little response from patients, but this expectation was partly attributable to the large number of poorly literate individuals in that particular practice.

## Discussion

During the patient interviews some problems with the questionnaire emerged, resulting in modifications. Most of the problems were in the category comprehension. Some words were changed or removed and some parts of a sentence were underlined for emphasis. A response category was added to two questions in order to overcome a recall problem. An open question asking about any other condition in the family was also added. Patients were happy with the length of the questionnaire, as also described in the study by Reid et al. [12]. The patients indicated that they would be willing to fill in the questionnaire if asked by their GP.

In the GP interviews two main themes arose: ‘the content of the questionnaire’ and ‘the use of the questionnaire’. Three terms were identified in the questionnaire which the GPs felt would be difficult for patients to understand: diabetes, heart disease and melanoma. The GPs were happy with the length of the questionnaire, and most thought that the questions were well-formulated. Most GPs agreed that the questionnaire covered important conditions, but they did miss an open question on rarer conditions. Some GPs suggested a different structure for the questionnaire.

At present, GPs only ask about family history when a patient consults regarding a problem related to an existing diagnosis. Family history is therefore applied passively, a conclusion that corresponds to the results of other studies [5, 20]. Family history plays a role in some consultations, but GPs in a previous focus group study stated that family history and pedigree drawing were not part of their daily routine [4]. Some GPs included a question on family history in their intake form for new patients. Most GPs considered the questionnaire useful in GP practice and felt that it would help them if available in a more proactive form. They would use it in risk management and thus apply genetics in prevention and the early detection of disease. The basic requirements for a useful questionnaire include a consistent position for family history in the EHR, accurate answers to questions (meaning that questions elicit the appropriate answers from patients) and a clear patient information leaflet explaining the questionnaire. Barriers mentioned were a lack of time and the practical implementation. In a previous publication we proposed a roadmap to stepwise integration of genetics in family medicine and clinical research. A validated questionnaire would mean that taking a family history would be possible within existing time constraints, and registration in EHRs would allow early identification of disease [7]. In addition, Wilson et al. [21] reported in 2016 on how repeated family history registration in EHR could support genetics in primary care and how theory informs professional education, writing “We illustrate how understanding psychological factors salient to behaviour can be used to tailor professional educational interventions”. Through exploratory study they found that family physician intentions were lower for “making a risk assessment” because this competency was perceived as more difficult than “taking a family history” and “making a referral decision”.

In our opinion, use of the Dutch family history questionnaire in daily practice could in theory help improve confidence in genetic skills through improved perceived behavioural control. Previously Daelemans et al. reported most GP participants interviewed said they had a lack of time as the main reason why they do not optimally record the family history. They noticed that the patient will probably not be keen to answering a long list of questions that is not related to their actual symptoms. The Dutch family history questionnaire could therefore potentially serve to overcome these obstacles for it is limited in length.

Making the questionnaire available in daily practice through eHealth applications could also serve to reinforce genetics because it is consistent with the GP’s subjective gatekeeper role [7]. GPs use national guidelines to stay up to date in timely diagnosis and treatment in busy daily practice. Incorporating the family history questionnaire through eHealth in EHR could result in timely identifying the family history as an additional risk factor and make actual improvement in and evaluation of genetic skills (such as assessing effective referral to the Department of Clinical Genetics) possible [27].

When the GP’s concerns are compared to patient problems with the questionnaire, some differences emerge. ‘Type 1 or type 2’ was indeed confusing for some patients, as anticipated by some GPs. ‘Melanoma’ was occasionally seen as a difficult term but its meaning was clear from the explanation in parentheses. Both groups were happy with the length of the questionnaire. As some GPs expected, ‘more than 1’ in questions 5 and 9 was indeed misunderstood. However, in contrast to the expectation of GPs, the length of the questions presented no problems for patients. GPs and patients both commented on the absence of an open question on other rarer diseases that might run in a family, and both felt that one additional option would suffice.

Emery et al. compared their family history screening questionnaire with a 3-generation pedigree that was prepared by a genetic counsellor. They found that the questionnaire shows good performance when screening primary care patients (among 526 patients, aged 20 to 50 years, in 6 general practices in Perth, western Australia) for increased disease risk due to family history [11]. Walter et al. performed a two-stage diagnostic validation study in 10 general practices in eastern England (stage 1: 618 patients; stage 2: 529 patients) comparable to Emery et al. although using a shorter questionnaire, and found comparable results regarding diagnostic accuracy of the questionnaire [23]. Emery et al. mentioned that the questions were tested in a pilot study but did not provide further details. Walter et al. mentioned that face validation was tested using a panel of lay members. Our study validated the translated Dutch family history questionnaire for face and content validity, the first step in the implementation of a Dutch family history questionnaire. Although the questionnaire is now validated for the Netherlands, the method used could be extrapolated to other medical systems internationally to enable wider implementation.

## Strengths and limitations

A strength of our study was the use of purposeful sampling to create a heterogeneous group of both patients and GPs. The cohort of patients interviewed however was mostly female and middle level of education, but age, gender and level of education is expected to be representative of most patients visiting the GP practice as registered in 2018 by the Statistics Netherlands’ database (6 female patients vs 4.5 male patient GP contacts a year in 2018; 29% of all patients registered received higher level education, 38% middle level education and 31% lower level education; patients 20–50 years of age visited the GP 4.6 a year vs age group 50–75 years 6.4 times a year) [28, 29]. Participant GPs interviewed are also expected to follow the registered GP data from 2016 according to Netherlands institute for health services research (NIVEL) [30]. 25% of all registered GPs (n = 9798) in the Netherlands according to NIVEL registry, were younger than 40 years of age, 51% were female and 67% worked part-time. 18% were registered to work in solopractice, 40% in duopractice and 42% in group practice, which is reasonably comparable with our relatively small GP cohort (respectively 2, 3 and 5 GPs).

Secondly, the analysis was carried out by two researchers independently. In depth analysis of the questionnaire was conducted by both patients and GPs, using two different methods. This is in contrast to studies by Wood et al. and Fuller et al. who confined their interviews to physicians and therefore overlooked patient perspectives [24, 25]. Furthermore, the questionnaire was improved over several rounds to create a prototype.

Although conclusions to this study are based on the opinions of a small group of GPs that may not be fully representative of all Dutch GPs, there was a wide range in age and practice experience. The researchers OH and EH who coded and analysed the interviews independently, made sure data saturation was reached, when no new information could be derived from the ten GP interviews. Future studies will need to affirm these conclusions.

Another limitation of the study was the distribution of educational levels. Only one patient with a lower education was included. As 28.6% of the Dutch population had only a lower education according to 2017 figures, our research population was less heterogeneous than the general population [22, 26]. Another potential limitation was a possible information bias due to the inclusion of four patients with a known genetic disease. However, this inclusion could also be considered a strength. Through the inclusion of these individuals in the design, we ensured a diverse array of patients would be able to understand the questionnaire.

## Conclusion

In a study in a Dutch primary care setting we validated an Australian family history questionnaire for face and content validity. We also explored the opinion of GPs regarding the questionnaire. Further research in a larger sample size in GP practice is needed to test the user-friendliness of the questionnaire and whether the barriers highlighted by the GPs would limit practical implementation. The effect on the prevention of the conditions covered in the questionnaire should also be studied in a larger sample size. Before the questionnaire can be implemented for early detection of chronic diseases in GP practice, the EHR must be updated to allow family history recordkeeping at a standardized retrievable location. The questionnaire can be used in GP practice as a screening tool, to discuss family history with patients, and to obtain a better risk assessment and possible timely referral to a department of Clinical Genetics. For those patients with an increased familial risk of common diseases based on the questionnaire, pre-symptomatic diagnostic tests and preventive treatment could be performed earlier, before related symptoms emerge.

## Appendices

1. Questionnaire family history v1.0
2. Interview guide General Practitioner
3. Interview guide patient
4. Questionnaire General Practitioner
5. Questionnaire patient
6. Questionnaire family history v5.0
7. Questionnaires and interview guides in Dutch

### Appendix 1: Questionnaire family history v1.0

1. Have any of your relatives (parents, brothers, sisters, children) had heart disease below the age of 60?
  - No
  - Yes
2. Have any of your relatives (parents, brothers, sisters, children) had diabetes (type 1 or type 2)?
  - No
  - Yes
3. Have any of your relatives (parents, brothers, sisters, children) had melanoma (malignant mole)?
  - No
  - Yes
4. Have any of your relatives (parents, brothers, sisters, children) had bowel cancer before the age of 55?
  - No
  - Yes
5. Do you have more than 1 relative (parents, children, brothers, sisters, grandparents, aunts, uncles, nieces, nephews and grandchildren) on the same side of the family who has had bowel cancer (independent of age)?
  - No
  - Yes
6. Have any of your male relatives (father, brothers, sons) had prostate cancer below the age of 60?
  - No
  - Yes
7. Have any of your female relatives (mother, sisters, daughters) had ovarian cancer?
  - No
  - Yes
8. Have any of your relatives (parents, brothers, sisters, children) had breast cancer below the age of 50?
  - No
  - Yes
9. Do you have more than 1 relative (parents, children, brothers, sisters, grandparents, aunts, uncles, nieces, nephews and grandchildren) on the same side of the family who has had breast cancer (independent of age)?
  - No
  - Yes

### Appendix 2: Interview guide General Practitioner

- First fill in the questionnaire for characteristics

Before starting the interview, the GP reads the questionnaire about family history, then the following questions are discussed with them:

- ***Do you think terminology is being used in the questionnaire that patients would not understand?***
- ***Do you think the questionnaire is useful?***
- ***What do you think of the number of questions?***
- ***Are there questions that you would like to modify?***
- ***Do you think any questions are missing from the questionnaire?***
- ***Could you see this questionnaire being used in everyday GP practice?***
  - ***If so, Why? How would you use the questionnaire?***
  - ***If not: Why not?***
- ***Are you already asking your patients all these questions?***
  - ***If yes: At what opportunity? Every patient? Who asks the questions?***
  - ***If not: Why not?***
- ***Do you have any additional comments concerning the questionnaire?***

### Appendix 3: Interview guide patient

- First complete the questionnaire for characteristics
- Next complete the questionnaire family history
- **What do you think defines a hereditary condition?**

Questions for each question in the questionnaire:

- ***Can you repeat the question in your own words?***
- ***How sure are you of your answer to the question?***
- ***Was it easy or difficult to answer the question? Why?***
- ***How did you arrive at the answer?***
- ***How would you like to improve this question?***

Question for question 1 of the questionnaire: ***What do you mean by ‘a heart condition’?***

Question for question 2 of the questionnaire: ***What do you mean by ‘diabetes’?***

Question for question 3 of the questionnaire: ***What do you mean by ‘a melanoma (malignant mole)’?***

General questions at the end:

- ***What would you think if your doctor used this questionnaire during your consultation?***
- ***What did you think of the number of questions?***
- ***Did you want to give answers other than yes or no?***
- ***Do you have any additional comments concerning the questionnaire?***

### Appendix 4: Questionnaire General Practitioner

#### Fill in before the interview

Age? ……… years old

What is your gender?

- □ Man
- □ Woman

What type of general practitioner are you?

- □ GP partner
- □ Sessional GP
- □ GP in training
- □ Other (please specify) …………………………………………………………….

At the moment you work in a:

- □ Solo practice
- □ Duo practice
- □ Group practice
- □ Health centre
- □ Other (please specify) …………………………………………………………….

What kind of area is the practice in?

- □ Urban
- □ Rural
- □ Other (please specify) …………………………………………………………….

### Appendix 5: Questionnaire patient

#### Fill in before the interview

Age? ……… years old

What is your gender?

- □ Man
- □ Woman

Level of education?

- □ No education
- □ Primary education
- □ Lower secondary education
- □ Lower vocational education
- □ Secondary vocational education
- □ High school
- □ Higher professional education
- □ University
- □ Other (please specify) …………………………………………………………….

Current Employment?

- □ Paid employment
- □ Entrepreneur
- □ Voluntary work
- □ Student
- □ Retired
- □ Unemployed
- □ Other (please specify) …………………………………………………………….

Do you have a genetic disorder?

- □ No
- □ Yes

### Appendix 6: Questionnaire family history v5.0

*Each question states which family members are referred to in that question. The question concerns your own family members, not family by marriage.*

1. Have any of your *close* relatives (parents, brothers, sisters, children) had heart disease *below the age of 60*?
  - No
  - Yes
2. Have any of your *close* relatives (parents, brothers, sisters, children) had diabetes?
  - No
  - Yes
3. Have any of your *close* relatives (parents, brothers, sisters, children) had melanoma (malignant mole)?
  - No
  - Yes
4. Have any of your relatives (parents, brothers, sisters, children) had bowel cancer *below the age of 55*?
  - No
  - Yes
5. Do you have *2 or more* relatives (parents, children, brothers, sisters, grandparents, aunts, uncles, nieces, nephews and grandchildren) *on the same side of the family* (*father’s*-*/mother’s side*) who have had bowel cancer (independent of age)?
  - No
  - Yes
  - *Not to my knowledge*
6. Have any of your male relatives (father, brothers, sons) had prostate cancer *below the age of 60*?
  - No
  - Yes
7. Have any of your female relatives (mother, sisters, daughters) had ovarian cancer?
  - No
  - Yes
8. Have any of your *close* relatives (parents, brothers, sisters, children) had breast cancer *below the age of 50*?
  - No
  - Yes
9. Do you have *2 or more* relatives (parents, children, brothers, sisters, grandparents, aunts, uncles, nieces, nephews and grandchildren) *on the same side of the family* (*father’s*-*/mother’s side*) who have had breast cancer (independent of age)?
  - No
  - Yes
10. *Are there any other conditions or illnesses running in your family?*
  - *No*
  - *Yes, please specify:…………………………*

### Appendix 7: Questionnaires and interview guides in Dutch

#### Vragenlijst erfelijkheid

1. Heeft een van uw familieleden (ouders, broers, zussen, kinderen) een hartaandoening gehad voor de leeftijd van 60 jaar?
  - Nee
  - Ja
2. Heeft een van uw familieleden (ouders, broers, zussen, kinderen) diabetes (type 1 of type 2)?
  - Nee
  - Ja
3. Heeft een van uw familieleden (ouders, broers, zussen, kinderen) een melanoom (kwaadaardige moedervlek) (gehad)?
  - Nee
  - Ja
4. Heeft een van uw familieleden (ouders, broers, zussen, kinderen) darmkanker gehad voor de leeftijd van 55 jaar?
  - Nee
  - Ja
5. Heeft u meer dan 1 familielid (ouders, broers, zussen, kinderen, grootouders, ooms, tantes, neven, nichten en kleinkinderen) aan dezelfde kant van de familie die darmkanker heeft gehad (onafhankelijk welke leeftijd)?
  - Nee
  - Ja
6. Heeft een van uw mannelijke familieleden (vader, broers, zoons) prostaatkanker (gehad) voor de leeftijd van 60 jaar?
  - Nee
  - Ja
7. Heeft een van uw vrouwelijke familieleden (moeder, zussen, dochters) eierstokkanker (gehad)?
  - Nee
  - Ja
8. Heeft een van uw familieleden (ouders, broers, zussen, kinderen) borstkanker gehad voor de leeftijd van 50 jaar?
  - Nee
  - Ja
9. Heeft u meer dan 1 familielid (ouders, broers, zussen, kinderen, grootouders, ooms, tantes, neven, nichten en kleinkinderen) aan dezelfde kant van de familie die borstkanker heeft gehad (onafhankelijk welke leeftijd)?
  - Nee
  - Ja

#### Interview schema huisarts

- Eerst algemene vragen laten invullen.

Voor het starten met het interview krijgt de huisarts de vragenlijst erfelijkheid te lezen. Vervolgens worden de volgende vragen met hen doorgenomen en hierbij doorgevraagd:

- ***Denkt u dat er termen in de vragenlijst staan die patiënten niet begrijpen?***
- ***Vindt u de vragenlijst nuttig?***
- ***Wat vindt u van de hoeveelheid vragen?***
- ***Zijn er vragen die u zou willen aanpassen?***
- ***Missen er volgens u vragen in de vragenlijst?***
- ***Ziet het gebruik van deze vragenlijst bij uw patiënten voor zich in de huisartsenpraktijk?***
  - ***Zo ja: Waarom wel? Op welke manier zou u de vragenlijst gebruiken?***
  - ***Zo nee: Waarom niet?***
- ***Stelt u deze vragen nu al aan al uw patiënten?***
  - ***Zo ja: Wanneer? Elke patiënt? Door wie worden de vragen gesteld?***
  - ***Zo nee: Waarom niet?***
- ***Heeft u verder nog verdere opmerkingen over de vragenlijst?***

#### Interview schema patiënt

- Eerst algemene vragen laten invullen.
- Vervolgens Vragenlijst erfelijkheid laten invullen.
- **Wat verstaat u onder een erfelijke aandoening?**

Vragen bij elke vraag van de vragenlijst:

- ***Kunt u de vraag in uw eigen woorden herhalen?***
- ***Hoe zeker bent u van uw antwoord op de vraag?***
- ***Was het moeilijk of makkelijk om de vraag te beantwoorden? Waarom?***
- ***Hoe kwam u op het antwoord?***
- ***Hoe zou u deze vraag nog willen verbeteren?***

Vraag bij vraag 1: ***Wat verstaat u onder ‘een hartaandoening’?***

Vraag bij vraag 2: ***Wat verstaat u onder ‘diabetes’?***

Vraag bij vraag 3: ***Wat verstaat u onder ‘een melanoom (kwaadaardige moedervlek)’?***

Algemene vragen aan het eind:

- ***Wat vindt u ervan als uw huisarts dit met u door zou nemen?***
- ***Wat vond u van de hoeveelheid vragen?***
- ***Had u andere antwoorden willen geven dan alleen ja of nee?***
- ***Heeft u verder nog opmerkingen over de vragenlijst?***

**Invullen voor interview:**

Leeftijd? ……… jaar

Wat is uw geslacht?

- □ Man
- □ Vrouw

Wat voor soort huisarts bent u?

- □ Huisartspraktijkhouder
- □ Huisarts in dienst van een andere huisarts
- □ Waarnemend huisarts
- □ Huisarts in opleiding
- □ Anders, namelijk …………………………………………………………….

U werkt op dit moment in een:

- □ Solopraktijk
- □ Duopraktijk
- □ Groepspraktijk
- □ Gezondheidscentrum
- □ Anders, namelijk …………………………………………………………….

Deze praktijk bevindt zich:

- □ In een stad
- □ Groot dorp
- □ Klein dorp
- □ Anders, namelijk …………………………………………………………….

**Invullen voor interview:**

Leeftijd? ……… jaar

Wat is uw geslacht?

- □ Man
- □ Vrouw

Wat is uw hoogst behaalde diploma?

- □ Geen opleiding
- □ Lager onderwijs
- □ Lager beroepsonderwijs
- □ Middelbaar algemeen voortgezet onderwijs
- □ Middelbaar beroepsonderwijs
- □ HAVO/ VWO
- □ HBO
- □ Universiteit
- □ Anders, namelijk …………………………………………………………….

Wat is uw huidige werk?

- □ Betaald in loondienst
- □ Ondernemer
- □ Vrijwilligerswerk
- □ Student
- □ Gepensioneerd
- □ Werkloos
- □ Anders, namelijk …………………………………………………………….

Heeft u een erfelijke aandoening?

- □ Nee
- □ Ja

**Vragenlijst erfelijkheid**

*In elke vraag staat benoemd om welke familieleden het in die vraag gaat. In de vragen gaat het om uw eigen familieleden, dus niet om aangetrouwde familieleden.*

1. Heeft een van uw *naaste* familieleden (ouders, broers, zussen, kinderen) een hartaandoening gehad *voor de leeftijd van 60 jaar*?
  - Nee
  - Ja
2. Heeft een van uw *naaste* familieleden (ouders, broers, zussen, kinderen) *suikerziekte*?
  - Nee
  - Ja
3. Heeft een van uw *naaste* familieleden (ouders, broers, zussen, kinderen) een melanoom (kwaadaardige moedervlek) (gehad)?
  - Nee
  - Ja
4. Heeft een van uw *naaste* familieleden (ouders, broers, zussen, kinderen) darmkanker gehad *voor de leeftijd van 55 jaar*?
  - Nee
  - Ja
5. Heeft u *2 of meer* familieleden (ouders, broers, zussen, kinderen, grootouders, ooms, tantes, neven, nichten en kleinkinderen) *aan dezelfde kant* *(vaders*-*/moederskant)* van de familie die darmkanker hebben gehad (onafhankelijk welke leeftijd)?
  - Nee
  - Ja
  - *Niet voor zo ver ik weet*
6. Heeft een van uw mannelijke *naaste* familieleden (vader, broers, zoons) prostaatkanker (gehad) *voor de leeftijd van 60 jaar*?
  - Nee
  - Ja
7. Heeft een van uw vrouwelijke *naaste* familieleden (moeder, zussen, dochters) eierstokkanker (gehad)?
  - Nee
  - Ja
8. Heeft een van uw *naaste* familieleden (ouders, broers, zussen, kinderen) borstkanker gehad *voor de leeftijd van 50 jaar*?
  - Nee
  - Ja
9. Heeft u *2 of meer* familieleden (ouders, broers, zussen, kinderen, grootouders, ooms, tantes, neven, nichten en kleinkinderen) *aan dezelfde kant* *(vaders*-*/moederskant)* van de familie die borstkanker hebben gehad (onafhankelijk welke leeftijd)?
  - Nee
  - Ja
  - *Niet voor zo ver ik weet*
10. *Komen er nog andere erfelijke aandoeningen voor in de familie?*
  - *Nee*
  - *Ja, namelijk …………………………………………………………………………………*

## Abbreviations

GP(s): general practitioner(s)
EHR: electronic health record
FaMe-net: Family Medicine Network, a merger of the Transition Project and Continuous Morbidity Registration Nijmegen, The Netherlands
CVA: cerebrovascular accident
